# Conducting an immersive community-based assessment of post-hurricane experience among Puerto Ricans: lived experience of medical ecology in an environmental disaster and migration

**DOI:** 10.1186/s12889-020-09735-w

**Published:** 2020-10-29

**Authors:** D. Vega Ocasio, J. G. Pérez Ramos, T. D. V. Dye

**Affiliations:** Department of Obstetrics and Gynecology, University of Rochester, School of Medicine and Dentistry, 601 Elmwood Avenue, Box 668, Rochester, NY 14642 USA

**Keywords:** Environment, Disaster, Migration, Hurricane María, Hurricane Irma, Community-based assessment, Medical ecology, Puerto Rico, Qualitative methods, Critical consciousness

## Abstract

**Background:**

Two devastating sequential hurricanes impacted Puerto Rico during September of 2017. The hurricanes were traumatic and created social and ecological upheaval throughout Puerto Rico, and subsequently in communities of Central Florida where affected Puerto Ricans migrated. The 2017 hurricane season exposed and exacerbated previous long-standing socio-political, economic, environmental, and health crises, generating a humanitarian emergency in the country. The consequences of these human-ecological disasters destroyed much of Puerto Rico’s residential and environmental infrastructure, displacing thousands of people and resulting in an unprecedented migration to the United States. We report on the lived experience of the investigator team and partnership in conducting community-based formative research subsequent to this disaster, research that aimed to identify salient issues relating to the impact of Hurricanes Irma and María on Puerto Rican communities both in Puerto Rico and in Central Florida.

**Discussion:**

The challenges faced during the conduct of this research include but are not limited to (1) emotional distress of participants and team members, (2) access to affected populations, and (3) precarious environmental factors, such as unstable infrastructure. To address these challenges, the researchers applied a Critical Medical Ecological paradigm along with qualitative methods to assist constructing explanatory models while obtaining internally-valid (from the community perspective), cathartic narrative accounts of the lived experience of hurricane survivors. The experience of the research team may help inform other investigators conducting applied research during a humanitarian crisis.

**Conclusion:**

Lessons learned in this research included: (1) usefulness of applying the Critical Medical Ecological model in the development of the project, (2) incorporating participation and methods that prioritize authenticity, (3) understanding the trauma experience and using study methods sensitive to it, and (4) innovating with best approaches to conduct the study given the challenges in post-hurricane Puerto Rico. These lessons could provide new insights on how to conduct in-depth participatory health research with community members who have been traumatized and – often – displaced. This research also demonstrates the value of pre-existing partnerships, critical consciousness in the field team, and medical ecological modeling as experiential for organizing complex, inter-related, multi-level variables that explain community and individual impact of environmental disasters.

## Background

The goal of this reflection is to analyze researchers’ experiences working in the context of a humanitarian crisis. We briefly describe the purpose of the study in the selected study site, the methods we employed, and lessons learned from the experience.

### Humanitarian context

During September 2017, Puerto Rico was hit by two devastating, sequential hurricanes (Irma and María). These two natural disasters exposed and exacerbated previous long-standing social-political, economic, environmental, and health crises in the country, generating an extreme humanitarian emergency, disrupting systems, communities, and causing widespread death and destruction [1–4]. For centuries, Puerto Rico has been exploited and colonized in ways that created vulnerability in its financial security, institutionalized inequitable trade policies, disenfranchised its voting populace, and created unsustainable dependencies [2, 5]. The lived experience of its citizens in advance of the hurricanes reflected both these vulnerabilities and resilience against adversity in Puerto Rico [6].

Hurricane María is considered to be the worst storm to strike the islands in decades, causing unprecedented damage and affecting the archipelago’s 3.4 million inhabitants [7]. The impact of this natural disaster disproportionately affected Puerto Rico’s most neglected and marginalized residents – with fewer resources available to help recover and rebuild – many of whom live in more rural and coastal communities and hard-to-reach mountainous areas [8]. The disaster left many communities stranded and without basic necessities including food, water, electricity, and cellphone services [9].

The University of Rochester (UR) has had a long-standing collaborative history with Puerto Rico for the past 20 years, most recently manifested as a collaborative CDC award (The CDC Global and Territorial Health Research Network) [10] that in 2015–2016 pursued community-based fieldwork focused around chronic disease [11, 12]. With the onset of the Zika outbreak in Puerto Rico in 2016, the University of Puerto Rico (UPR) –UR team rapidly shifted focus to community experiences and participation in Zika-related research [13, 14], culminating in a technology-centered comprehensive pilot project, termed mZAP (móvil Zonas, Acción y Protección) [14, 15]. With the unexpected occurrence of these two devastating hurricanes in 2017, the joint UPR-UR team abruptly ceased all research activity and transitioned to the creation of a volunteer community assistance strategy (People-to-People for Puerto Rico (#p2p4PUR)), mobilizing mobile clinic gear and teams in communities where UPR-UR had prior experience and in other communities where assistance was needed [16, 17]. With recovery in communities underway in 2018, the UPR-UR team resumed and shifted its research focus to document the disaster experience in Puerto Rican communities and among the diaspora community that left the island for the USA.

Natural disasters, such as Hurricane María, can cause significant socio-economic and physical damage to fragile environments and to susceptible populations, effects that can spread beyond the immediate locality [18]. In the case of Puerto Rico, the archipelago was already suffering from a political and economic crisis: the poverty level increased to 44.5%, the infrastructure (including the health care infrastructure) weakened, the economic recession resulted in austerity measures, and the impact from Hurricane Irma two weeks prior to Hurricane María caused a partial collapse of the electrical system [19, 20]. The island’s economic crisis - already impacting many households - fueled a sense of uncertainty and despair among many Puerto Ricans [21]. The 2017 hurricane season destroyed much of Puerto Rico’s infrastructure (including residential structures) and shifted natural resources as the landscape experienced visible change [22]. Thousands of properties became uninhabitable with communities upended, leading some residents to relocate within and outside of Puerto Rico [23], while others remained living in fragile dwellings [5, 24, 25]. The complex impact that this disaster had on the overall health and disease of the population, survival, and adaptation has yet to be fully documented [23, 26–29].

The purpose of this paper is to describe the lived experience of the investigators who conducted this work, presented as a case study, to draw recommendations for other researchers who may need to conduct research in times of humanitarian disaster.

#### Lesson 1: using the critical medical ecological model helped organize our work and response

Working amidst the devastation of the hurricanes, an appropriate framework needed to be implemented to capture the complex human-ecological calamity that resulted from the mentioned natural disasters. The Critical Medical Ecological paradigm [22, 30] provided a useful heuristic device to examine human interactions with their environment, comprehensively defined to include physical, social, political, and biological elements [31–33]. This analytical framework prioritizes multidimensional social (including economic, demographic, and cultural) relationships and biological aspects of community members while acknowledging the interdependent role of bioenvironmental factors and their associations with disease [34]. The model helped us prepare the research questions and fieldwork through thinking about how humans are influenced by multi-level ecosystems of which they are part: including the macro-micro ecosystems, the abiotic (e.g., floods) and biotic components (e.g., immune system responses, stress hormones), and multiple levels of human organization. In this project, the Critical Medical Ecological framework additionally drove both the aid/relief response [17] and subsequent research with Puerto Ricans and the diaspora, with the team immersing in the midst of post-hurricane communities to gather, first-hand, qualitative experiences from community members. The study aimed to provide a comprehensive approach to better understand the amalgam of factors that impacted Puerto Ricans through the post-hurricane experience, including their decisions - if made - to depart the islands.

#### Lesson 2: incorporation of participation and methods that prioritize authenticity

Investigators recognized the need to approach our research participants as collaborators and to strive toward building and relying upon authentic partnerships to collect and analyze data. We sought to build a grounded, community-based ecological model of coping and response to surviving Hurricanes Irma and María, including identifying determinants of and responses to migration from Puerto Rico to the continental USA. From our previous aid relief efforts in Puerto Rico with #p2p4PUR, we understood that qualitative methods were required to capture the range and nuance of the “lived experience” subsequently expressed by community members. Qualitative methods present the opportunity to capture natural, insider perspectives which – oftentimes - prove cathartic for participants [35–37].

To achieve community authenticity in our approach (methodologically, to improve validity), the qualitative interview guide was shaped by the Critical Medical Ecology paradigm and was, in part, based on the team’s prior experience in conducting qualitative work in Puerto Rico around Zika [13]. The interview guide was organized sequentially to walk the participants through their experience from pre- to post-hurricane, with partially structured questions that were focused yet allowed for elaboration, explanation, and interpretation through the interview’s conversation. Additional questions/ themes were included based on informal conversations around priorities with project partners. The final themes covered included participants’ peri-hurricane experiences, the social-ecological damage, health impact, reactions, and migration decision-making.

The project was reviewed and approved by 12 community groups, agencies, and organizations in Puerto Rico and Orlando. Prior to conducting the research, participants received an information sheet, and provided verbal consent for audio-recording. Field notes were taken by interviewers during and immediately after each interview to facilitate a more comprehensive analysis process. Moreover, a professional transcription firm was contracted to transcribe audio recordings to text, which were subsequently reviewed and edited by native Spanish speakers on the team. Importantly, to maintain research fidelity and validity, all research material was maintained, collected, and analyzed in Spanish by an exclusively bilingual Latinx, Spanish-fluent team. Study communities were selected from areas familiar to the team through previous research collaboration (that occurred prior to the hurricanes), and in communities the team became familiar with through its relief work (#p2p4PUR).

The study data was collected from 97 Puerto Rican participants (65 female and 32 male). In Puerto Rico, the study was conducted in nine municipalities around the archipelago encompassing different ecological zones (mountainous, metropolitan, rural, and outer island) to capture a range of ecological exposures for a total of 23 interview instances (17 focus groups and 6 individual interviews) among community members. In Florida, the study was conducted in Orlando (a major resettlement hub for post-hurricane Puerto Ricans) with a total of 27 participants (21 females and 6 males) in four focus groups. The Orlando groups covered the same topics as the Puerto Rico-based groups, but additionally focused on the post-hurricane Puerto Rican diaspora that migrated to the USA and their challenges associated with relocation.

Analysis prioritized community voices through iterative thematic analysis [38] and note-taking facilitated a preliminary understanding of salient community issues. Transcripts were uploaded to Dedoose (Socio-Cultural Research Consultants, Manhattan Beach, CA, v.8.3), a web-based qualitative data analytical software, which helped researchers synthesize multiple levels of qualitative data into a single platform, and enabled multi-layered and meaningful analysis [39]. The team iteratively developed a codebook that captured both the emic (community perspective) experience and also selective etic (research perspective from outside the culture) analytic codes required for analysis. A rigorous and extensive coding verification process by two native Spanish speaking team members was established, which included debriefing and peer validation of coding. The final coding process was completed by four native Spanish-speaking team members using the transcribed interviews.

#### Lesson 3: understand the trauma experience and incorporate study methods sensitive to it

Given the massive trauma experienced by Puerto Ricans in this period, and our witness to that suffering, we needed to prioritize understanding such experiences and incorporate it into our approach. This research study was able to observe and describe the complexity of the pre- and post-hurricane situation impacting Puerto Ricans. The results showed (summarized in Table 1) that the precarious conditions that followed Hurricane María were the result of a failed complex system that covers every layer of the Critical Medical Ecological model (sociocultural, biological, healthcare and abiotic): geographical areas, governmental response, financial limitations, individual decisions, inequitable social relationships, and a foreseen failure of the healthcare system. As explained in the model, Hurricane María had an effect from the community level to the individual level, for example complete communities were destroyed by the forces of the wind and many residents were affected, individually, by those repercussions. In many cases, the post-hurricane conditions increased or exacerbated social determinants of health that led to complicated health outcomes (increased comorbidities associated with pre-existing health conditions, restricted access to food and water, and death). Indeed, presenting “death narratives” was one of the most common themes in our project:

**Table 1:** Critical Medical Ecological Model: Identifying the broader factors that led to the precarious conditions before, during, and after the hurricane’s impact

	Sociocultural	Biological	Health Care	Abiotic
Community	Systems collapse (Water, electricity, food) Disbalanced social and political relationships Unemployment Poverty Limited access to and poor distribution of financial resources Precarious infrastructure Late federal/state response External dependency	Infectious disease exposure (e.g. leptospirosis) Shifting breeding grounds for vectors Bacterial, flora, fauna change	Collapse of medical facilities Limited health care personnel Only one trauma center in the island Limited medicines available Poor surveillance Traumatized health workers	Mountains Wind Rain Flood Debris Temperatures and humidity Mudslides Elevation Poor roads and bridges
Household	No communication Weak building construction Lack of home ownership documentation Family income Gender roles Migration/relocation Poor access to available aid and resources Social and family networks disrupted	Exposure to Infectious Diseases Stress/Trauma Impact of trauma in chronic diseases Increases in household risk Increase in vector and vermin exposures Crop loss/ loss of food sources	Isolation (unable to access medical facilities) Limited assistance for bedridden family members No reliable access to medication and treatment New, important social determinants emerge	Housing Damage Loss of material goods, appliances, and equipment
Individual	Unemployment Denial about hurricane Isolation Loneliness, isolation, depression, and anxiety Loss of social and family relationships Loss of role identification	Pre-existing medical conditions Weaken immune system Stress response Injuries	Unable to access healthcare facilities or treatments Stigma related to mental health care Loss of medication and therapeutic interventions	Lack of Protective clothing/footwear Sleeping environment disturbed

**“*Sí, luego del huracán, llegó, llegó mucha depresión en muchas personas, muchas historias que se escuchan porque mientras uno hace fila, uno conoce gente, y uno escucha tanta historia, que yo creo que la depresión de muchas personas de haber perdido todo fue uno de los mayores problemas, y a base de la depresión viene el suicidio, y se escucharon muchos casos en Puerto Rico de personas no solo que se enfermaron, también optaron, tomaron la decisión de suicidarse …*”** (“Yes, after the hurricane, there was a lot of depression in many people, you hear many stories while waiting on lines, you meet people, and you hear so many stories, that I believe that the depression of many people resulted by having lost everything, it was one of the bigger problems, and with depression comes suicide, and many cases were heard in Puerto Rico of people not only who got sick, they also chose, they took the decision to commit suicide ... ”

– Female participant in rural focus group

The complexity of these societal challenges, and how they made an impact at an individual level, resulted in the displacement and migration of many Puerto Ricans to the continental United States. Factors mentioned influencing this decision include: (1) unemployment as a result of the hurricane, (2) loss of household materials, and (3) medical needs such as dialysis and cancer treatment. The Critical Medical Ecological model allowed us to identify broader contexts of the situations that led to such precarious conditions before, during, and after the hurricane’s impact. The model permitted us to organize and to understand how previous social-environmental challenges influenced the fragile conditions in the aftermath - such as the long-standing humanitarian-financial crises that restricted families’ abilities to purchase goods, or the perceived confidence in community members due to a lack of recent experience with hurricanes.

Hurricane Irma and especially Hurricane María, presented traumatic events that subsequently created social and ecological upheaval throughout Puerto Rico, and in the diaspora communities of Central Florida (and elsewhere) where Puerto Ricans migrated. The consequential effects of this event and the challenges that followed were multifactorial, complex, and reflected interdependent social and health problems in an ecologically destructive context. Hence, the medical ecological paradigm and qualitative methods helped constructively build explanatory models while obtaining emic (and somewhat therapeutic) narrative accounts of the lived experience of hurricane survivors.

### Emotional distress of participants

Many participants did not have the opportunity to talk about their experiences previously, creating both challenges for the team (emotional sensitivity to the suffering of others) and research needs (to elicit first-hand accounts of a disastrous event from those who experienced it). Occasionally, the traumatic events that individuals experienced led to feelings of despair, sadness, and anxiety among participants. Prior to conducting field work, the team contacted local mental health institutions and organizations in each of the respective towns to identify referral information if needed during the interview process. All participants received a pamphlet that included the contact information for mental health assistance, and an island-wide helpline. Organizing partners who hosted data collection instances were best-suited to refer participants to local resources before, during, and after the period of this study.

### Emotional sensitivity of team members

The team encountered the stories of the struggles that participants experienced; their survival narratives and the effect these had both physically and mentally in the participants’ lives challenged the team’s capacity to remain both objective and empathetic in conducting this research. All three co-PIs of this study had relatives impacted in Puerto Rico from the hurricanes – this event was a personal tragedy for all involved, yet an important, action-driven scientific opportunity to potentially learn local strategies that could reduce the damaging impact of future natural disasters.

**“*Esta es la primera vez que alguien nos pregunta como fue nuestra experiencia con el huracán*.”** (“This is the first time someone ask us about how our experience with the hurricane was.”)

Male participant, Rural Area, Focus Group

The daily debriefing sessions among team members not only served to discuss major learnings, but it also served as an informal therapeutic session to discuss well-being and mental health needs, as required.

#### Lesson 4: we innovated best approaches to create and conduct this study given the physical, social, and technical challenges in post-hurricane Puerto Rico

We had to develop innovative methods of data collection, such as leveraging pre-existing community networks from our other work in Puerto Rico, relying upon social media to access communications, and logistical planning sensitive to roads, weather, and timing.The ecological damage in the aftermath of the hurricanes presented logistical difficulties in reaching susceptible communities due to unstable infrastructure, such as poor road conditions, lack of electricity, and minimal communication. As part of the recruitment challenges - because of the limited communication infrastructures damaged by Hurricane María - the research group used different strategies to communicate with community partners, in particular social media networks such as WhatsApp and Facebook. The research team divided the island into quadrants to facilitate logistics and to ensure geographic coverage, prioritizing the communities that received humanitarian help previously from the research team.

Power outages and cellphone disruptions occurred due to the unstable infrastructure post-hurricane while completing the interview process. Road conditions made traditional routes inaccessible and continuing rain caused the team to monitor flood levels in their route. The research team worked together with community members to conduct interviews during times when it was more convenient to the community (typically during daylight and outside). Further, logistics for interview events were also developed in collaboration with community leaders beforehand, including mapping routes and locations (e.g., town hall, town bakery).

The challenges presented showcase the significant environmental and social risks that can follow natural disasters and emergency situations. Such natural hazards are likely to trigger additional challenges for both the researchers and the participants, perhaps limiting their ability to access certain communities, or limiting ability of potential participants to reach the meeting place. Recognizing the scale of disaster, and the impact it had on the population at a physical and psychological level is important for ensuring an effective response, insights informed by the team’s humanitarian efforts that preceded this research.

### Community partnership

This project was built on those community partnerships where the team has developed visibility and trust, either arising from the pre-hurricane research projects with communities throughout the island, or through subsequent experience with new communities through the #p2p4PUR relief response. Research partnerships in Orlando, however, did not pre-exist the hurricanes. As this research project emerged, the UR team networked to reach city officials and non-profit organizations focused on assisting Puerto Ricans settling in Orlando and community groups serving and advocating for various needs of the resettled Puerto Rican population. The team’s prior work in Puerto Rico added to their credibility to undertake this work and provided an opportunity for close collaboration with diaspora leaders.

The selection of the research team for this work (and for the collaborative research that preceded the hurricanes) was deliberate and crucial, improving validity and potential for action by prioritizing critical consciousness (Paulo Freire’s concept of *conscientização* [40]*)*, collecting and analyzing participants “lived experience” through a research team with strong connection to Puerto Rico, its history, culture, practices, and its contemporary social-political situation.

#### Scientific contribution

Puerto Rico’s history has resulted in multiple forms of social determinants of health that created social inequalities including lack of political representation (primarily in the USA), unfair workforce practices, institutionalized social control, racism, corruption, gender-based violence, poverty, and health discrimination [5, 41]. Given the ongoing austerity measures imposed on the country, the dual impact of the 2017 hurricanes have had an especially harsh impact on Puerto Rican communities [41]. The devastating, cumulative consequences of the hurricanes resulted in both an ecological and a multi-level social shift within the individual, household, and community levels that collapsed the medical infrastructure, exacerbated chronic diseases, increased infectious diseases, impacted mental health rapidly in community members, and created a significant migration to the continental United States [5]. Nonetheless, the damaging effects of these hurricanes are also a result of previous socio-economic and political challenges affecting the island.

Typically, natural disasters result in significant loss and damage leading most scientific research studies to focus on the health outcomes and risk factors that are presented in the wake of such an event. Such studies, however, subsequently often fail to evaluate the previous conditions and behaviors that may contribute to these post-disaster impacts. Understanding the social-ecological landscape before the natural disaster, however, provides a deeper understanding of the level of vulnerabilities and strengths in communities, and the complexities of how individuals perceive and respond to risks. The complex situation in Puerto Rico - together with the unprecedented damage caused by this natural disaster - made visible the need to evaluate and analyze the contributing roles of the physical and social-environmental circumstances of families, and the underlying factors contributing to the rapid collapse in living conditions in the aftermath of the hurricane. Consequently, this study sought to explore and answer questions concerning the type of services required to meet community needs, actions necessary to make programs or services more effective, and strategies required to overcome newly-defined problems, all from a community-centered perspective. This perspective is intended to assist stakeholders in developing future policies and plans aimed at addressing risk factors that contribute to a community’s susceptibilities in a natural disaster. This type of research is essential to offer policy-makers specific evidence grounded in the experiences of those most likely impacted by policy decisions (e.g., community members), taking into account related cultural, social, political, and economic realities [42]. Furthermore, the results from this field work are expected to assist community leaders in the understanding of the risk factors associated with natural disasters (specific to their community), community-driven methods of organization, and how community engagement is an integral part of response from the onset of the emergency. The research team intends to continue collaboration with participant leaders and to report back the results of this work.

## Conclusion

This case study sought to describe salient issues and factors relating to conducting research on the impact of Hurricanes Irma and María in Puerto Rican communities - both in Puerto Rico and Central Florida – on how ecological risks may have changed, their impacts on health, and the responses and determinants for relocation. Crucial among the lessons learned are that researchers needed to be sensitive to, and ready for, recollecting negative, emotional events, and to allow the team (scientifically) to express that emotion. The data collected during this research will serve future studies as best practices for community-engagement and to address data collection rapidly, post-disaster. The importance of collecting and disseminating this research allows scientific teams to learn about the intricacies and challenges associated with a natural disaster within a complex medical ecological theoretical framework and to potentially prepare prevention and remediation mechanisms for future events.

In addition, researchers need to become acquainted with the risks and benefits for such types of methods (for example, the lengthy time required to properly code qualitative data and analyze with validity) and the issues of importance prior to engaging communities, understanding the potential harms for each specific setting, and preparing to manage possibly daunting situations. Seeing the community as true partners in collectively documenting and voicing their experiences helps leverage the strengths of the methods and creates a mutually-supportive research environment. Although communities were not expected to have a direct benefit from these experiences as our preliminary findings suggest, community participants describe the therapeutic effects of being allowed to talk about their experiences. As previous research has demonstrated, the importance of a social support system after a traumatic event helps cope with the distress occurring during the emergency [32]. Engaging with respective stakeholders and community leaders prior to the start of and after the study is vital for success.

Because of our commitment to Freirean community principles of *conscientização*, equity, and respect, this research study provided an environment of respect and trust, where community members’ voices were heard and prioritized. Additionally, having collaborated previously with stakeholders and community members facilitated the process of respect and trust. The team was predominantly Puerto Rican – all with prior experience in Puerto Rico - collecting and analyzing data in local parlance without hierarchical power dynamics guiding our team’s actions. This deliberate lack of a hierarchical structure ensured that participants and researchers co-existed in an environment of cultural respect and understanding. This respect represented an significant lesson among the researchers because it provided a deeper understanding of the importance in creating meaningful connections with participants beyond the research needs and - in this example - before the disaster even occurred. Finally, reporting results back to the community in a timely manner, obtaining feedback, and refining conclusions, is a critical component of this process that helps bring closure to this phase of the work.

## Data Availability

No data is available since the paper is on our experience and as qualitative data for the main study is identifying, we will not make it publicly available.

## Abbreviations

CDC: Center for Disease Control and Prevention
UPR: University of Puerto Rico
UR: University of Rochester
mZAP: Zonas, Acción y Protección
#p2p4PUR: People-to-People for Puerto Rico
RQI: Rapid Qualitative Inquiry
